# Heparan Sulfate Biosynthesis: Methods for Investigation of the Heparanosome

**DOI:** 10.1369/0022155412460056

**Published:** 2012-12

**Authors:** Hinke A. B. Multhaupt, John R. Couchman

**Affiliations:** Department of Biomedical Sciences, University of Copenhagen, Copenhagen, Denmark

**Keywords:** glycosaminoglycan, proteoglycan, heparin, Golgi apparatus, confocal microscopy

## Abstract

Heparan sulfate is perhaps the most complex polysaccharide known from animals. The basic repeating disaccharide is extensively modified by sulfation and uronic acid epimerization. Despite this, the fine structure of heparan sulfate is remarkably consistent with a particular cell type. This suggests that the synthesis of heparan sulfate is tightly controlled. Although genomics has identified the enzymes involved in glycosaminoglycan synthesis in a number of vertebrates and invertebrates, the regulation of the process is not understood. Moreover, the localization of the various enzymes in the Golgi apparatus has not been carried out in a detailed way using high-resolution microscopy. We have begun this process, using well-known markers for the various Golgi compartments, coupled with the use of characterized antibodies and cDNA expression. Laser scanning confocal microscopy coupled with line scanning provides high-quality resolution of the distribution of enzymes. The EXT2 protein, which when combined as heterodimers with EXT1 comprises the major polymerase in heparan sulfate synthesis, has been studied in depth. All the data are consistent with a cis-Golgi distribution and provide a starting point to establish whether all the enzymes are clustered in a multimolecular complex or are distributed through the various compartments of the Golgi apparatus.

Heparan sulfate is probably the most complex carbohydrate of the Bilateria. This arises not from the structure of the basic polymer, which is a repeating disaccharide, but from the further modifications, most notably sulfation. All multicellular animals possess heparan sulfate, and genetic experiments in invertebrates such as *Caenorhabditis elegans*, as well as the mouse, show that it is indispensable for life (Lin et al. 2000; Kitagawa et al. 2007). Almost certainly this derives from its ability to interact with a plethora of ligands belonging to many protein families (Esko and Selleck 2002). Examples from growth factors, chemokines, cytokines, morphogens, extracellular matrix glycoproteins and collagens, enzymes as diverse as metzincins, and lipases are recorded (Esko and Selleck 2002; Bishop et al. 2007; Couchman 2010). Moreover, a diverse array of pathogens use heparan sulfate to gain entry to cells (Bartlett and Park 2010).

Despite the wide array of ligands, the number of core proteins that carry heparan sulfate chains is rather few. Two major families of heparan sulfate proteoglycans (HSPGs) are the cell surface glypicans and syndecans. Others are a small unrelated set of proteins such as type XVIII collagen (Seppinen and Pihlajaniemi 2011), agrin, and perlecan (Iozzo et al. 2009) that are found in many basement membranes and other extracellular matrices. Cell surface betaglycan and neuropilin-1 may also have a single heparan sulfate chain (Couchman 2010), whereas a specific splice variant of the hyaluronan receptor CD44 can also be substituted with this glycosaminoglycan (Puré and Assoian 2009). With so many ligands, yet so few HSPGs, it appears that the major roles of heparan sulfate are to concentrate ligands or control their gradients within tissues (Lander and Selleck 2000; Bishop et al. 2007). Where transmembrane signaling is involved (e.g., in the syndecans), then presumably a wide variety of incoming stimuli can trigger a conservative number of downstream events. Often cell surface proteoglycans work in concert with high-affinity receptors (Alexopoulou et al. 2007; Polanska et al. 2009; Xian et al. 2010). The best examples are known from fibroblast growth factor (FGF), and crystal structures are recorded for the ternary complexes of FGF, heparin, and FGF receptor (Pellegrini et al. 2000; Schlessinger et al. 2000).

## Heparan Sulfate Structure and Synthesis

**Figure 1.**
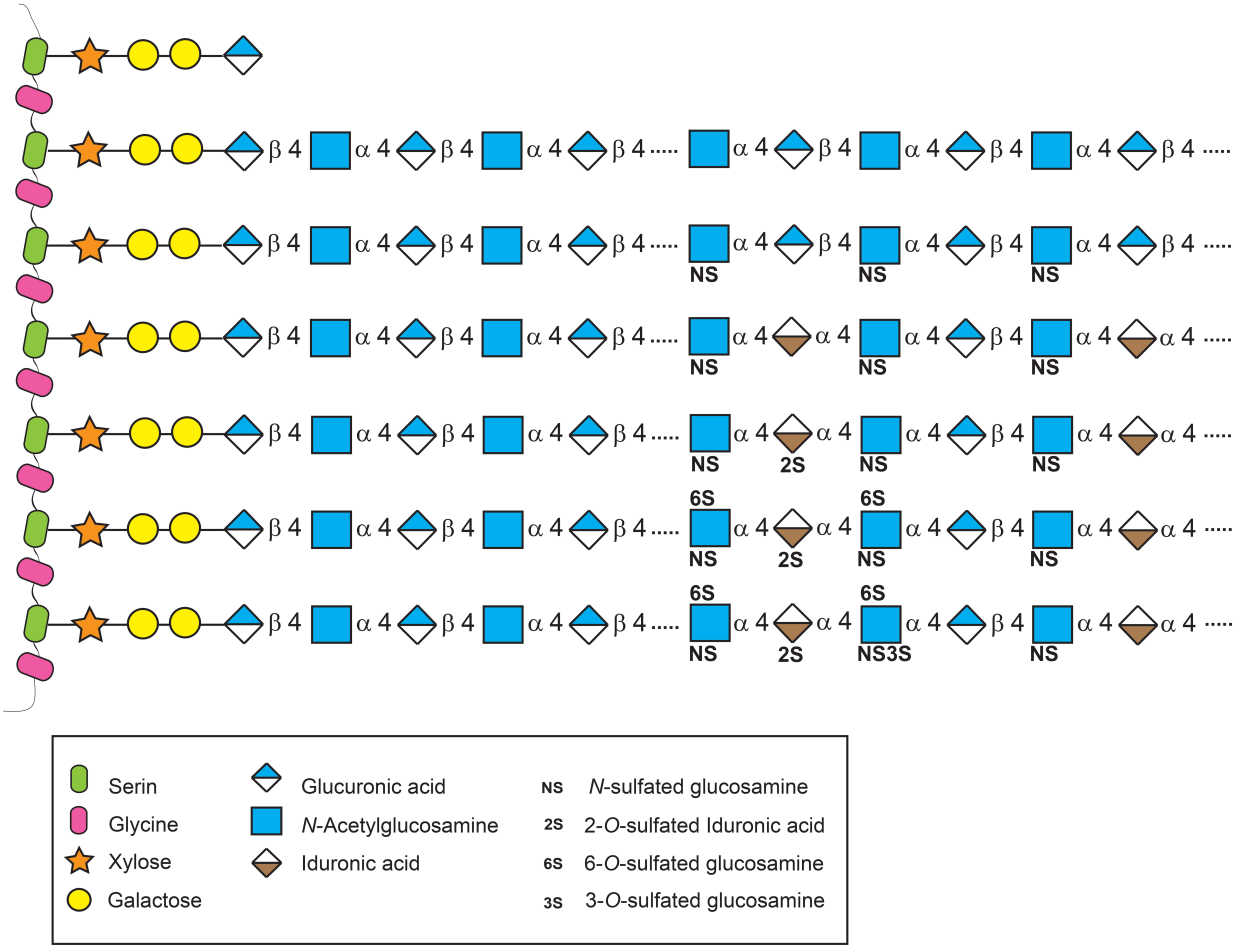
Schematic representation of heparan sulfate synthesis and its modifications. Heparan sulfates are sugar chains that consist of repeated disaccharides linked to serine residues on a protein core through the sequence xylose–galactose–galactose–uronic acid. Two polymerases, EXT1 and EXT2, are responsible for the elongation of the chain. The disaccharide residues are further modified by sulfotransferases and epimerase to obtain a mature glycosaminoglycan. However, these modifications do not go to completion, resulting in domains of high, intermediate, and low sulfation, enabling the generation of many potential heparan sulfate structures and therefore ligand-binding sites.

The composition of heparan sulfate from a variety of tissues across a number of species is well documented, although perhaps not as well known as those from invertebrates where traditional biochemical approaches are more demanding. Moreover, the sequences of all the enzymes that contribute to heparan sulfate biosynthesis are catalogued from a number of genomes across the animal kingdom. However, the sheer complexity of heparan sulfate structures poses interesting questions and problems regarding synthesis. Initiation is characterized by the transfer of xylose to a serine acceptor on the core protein. This is followed by two galactose units and a glucuronic acid moiety. The completed tetrasaccharide is often referred to as a stem or linker, because it is common to heparan sulfate and chondroitin/dermatan sulfate synthesis (Couchman and Pataki 2012; Fig. 1). In the case of heparan sulfate, repeating disaccharides of *N*-acetylglucosamine and glucuronic acid are added, and in some cases, 50 disaccharides or more may follow. The polymerase consists of two proteins, EXT1 and EXT2, that form heterodimeric complexes (McCormick et al. 2000). Data suggest that before chain elongation is completed, early modification steps occur. The first is *N*-deacetylation and *N*-sulfation, carried out by one of four *N*-deacetylases/*N*-sulfotransferases (NDSTs) (in mammals). Both steps are carried out by a single protein. This is followed by epimerization of uronic acid residues, converting some of them from glucuronic to iduronic acid. Some of the iduronate is then sulfated at the 2-*O* position. In contrast to the NDSTs, there is a single 5′ epimerase and 2-*O*-sulfotransferase, reportedly forming a complex with each other (Pinhal et al. 2001). Next, 6-*O*-sulfation of glucosamine residues can occur; in mammals, three enzymes are capable of this modification (HS6ST-1, -2, and -3). Finally, and rarely, 3-*O*-sulfation takes place, but interestingly, seven mammalian enzymes are capable of completing this step (Esko and Selleck 2002).

In the case of heparin, characteristic of mucosal mast cell granules, chain modification is extensive, so that trisulfated disaccharides can be abundant (Casu et al. 1981; Björk et al. 1982; Tovar et al. 2012). However, adjacent to the core protein (serglycin; Kolset et al. 2012; Rönnberg et al. 2012), there is a sulfate-poor region, and this appears to be common to all HSPGs (Murphy et al. 2004). In contrast to heparin, however, heparan sulfate of syndecans and glypicans, for example, is not so extensively modified. Regions of low or no sulfation are interspersed between regions of high sulfation, and at the junctions between these zones are regions of intermediate sulfation (Murphy et al. 2004). Given that the overall pattern of chain modification is held relatively constant within a particular cell type but may differ between cell types, the control of heparan sulfate synthesis is clearly complex. Because it is not random, modifications must be regulated. For example, it is known that liver-derived heparan sulfate is more highly sulfated than that of other organs (Lyon et al. 1994; Nagamine et al. 2012). Still, today, there is little information regarding how cells control the pattern of chain modification. It is known that the activity of NDSTs lay down a template because *N*-sulfation largely determines where further modifications, such as epimerization and 2-*O*-sulfation, occur (Murphy et al. 2004; Kreuger and Kjellén 2012). However, even where NDST1 and NDST2 are deleted, some 6-*O*-sulfation takes place (Holmborn et al. 2004), even though *N*-sulfation may be absent.

## Localization of Heparan Sulfate Synthetic Machinery

**Figure 2.**
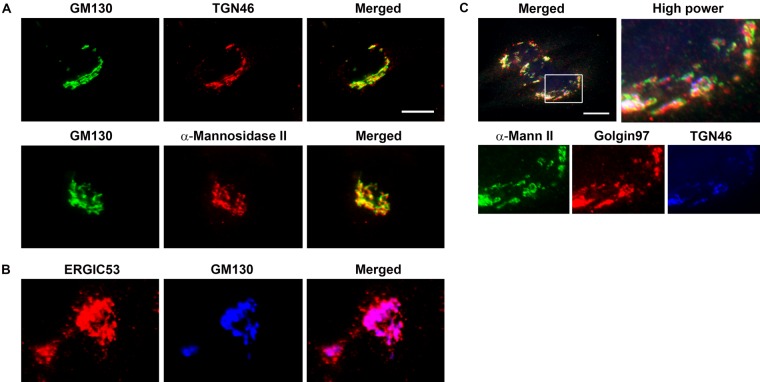
The Golgi compartments can be resolved using specific antibodies. Normal rat kidney (NRK) (A, B) and MDA-MB231 cells (C) were stained with antibodies specific for the cis-Golgi compartment (GM130), medial-Golgi (α-mannosidase II), trans-Golgi (Golgin97), and trans-Golgi network (TGN46). The endoplasmic reticulum (ER)–Golgi intermediate compartment was identified using the ERGIC53 antibodies. (A) GM130/TGN46 and GM130/α-mannosidase II double staining shows compartment discrimination. (B) Double staining for ERGIC53 and GM130 shows overlap of the intermediate ER-Golgi compartment with cis-Golgi, with the ERGIC53 extending more toward the ER. (C) Triple staining for α-mannosidase II, Golgin97, and TGN46 resolved the late compartments of the Golgi. Scale bar: 10 µm.

Despite the fact that genomics and biochemical analysis have provided details of the enzymes involved in heparan sulfate synthesis, and the products of their activity are increasingly well understood, little is known regarding the location of the enzymes. Early work suggested that xylosyltransferases were present in the endoplasmic reticulum or early Golgi (Vertel et al. 1993; Schön et al. 2006). The EXT enzymes, 5′ epimerase, NDST1, and other sulfotransferases have all been proposed as Golgi enzymes (McCormick et al. 2000; Crawford et al. 2001; Nagai et al. 2004; Busse et al. 2007), but high-resolution light or electron microscopic localization has not been performed in any case. However, light microscopic examinations are not simple, because as described by Dejgaard et al. (2007), it is important to carry out multiple localizations, coupled with laser scanning confocal microscopy and line scans of the stained material. Only in this way can increased certainty be obtained regarding the assignment of a Golgi enzyme to a particular compartment or “stack.” The Golgi can be divided into *cis, medial*, and *trans*, with the more dispersed, vesicular trans-Golgi network as a site for packaging cell surface and matrix components for export. There is also an endoplasmic reticulum/Golgi vesicular compartment on the cis face, termed ERGIC (Schweitzer et al. 1988; Saraste and Svensson 1991). To aid in localization of these domains, there are fortunately well-described specific antibodies that detect resident proteins (Table 1). As can be seen in Fig. 2, these antibodies stain discrete compartments within the Golgi, and these can be discriminated in double and triple staining protocols, using confocal immunocytochemistry. Some cells are much more amenable to Golgi studies than others, and among those most commonly used are normal rat kidney (NRK) cells, which are flat in culture with conspicuous and well-demarcated Golgi apparatus.

**Table 1.** Golgi Markers and EXT2 Protein Antibody Data

Antibody	Target	Dilution	Company
Rabbit anti-ERGIC53/p58	Endoplasmic reticulum (ER)–Golgi intermediate compartment	1:100	Sigma-Aldrich (St. Louis, MO)
Mouse anti-GM130	cis-Golgi	1:600	BD Transduction Laboratories (Franklin Lakes, NJ)
Rabbit anti–α-mannosidase II	medial-Golgi	1:200	Chemicon International (Temecula, CA)
Mouse anti-Golgin97	trans-Golgi	1:300	Molecular Probes (Eugene, OR), Invitrogen (Carlsbad, CA)
Sheep anti-TGN46	trans-Golgi network (TGN)	1:1000	Serotec (Kidlington, UK)
Goat anti-EXT2 (N-15 + C-17)	EXT2 protein	1:100	Santa Cruz Biotechnology (Santa Cruz, CA)

## Localization of Heparan Sulfate Biosynthetic Enzymes

It is of interest to localize the heparan sulfate synthetic enzymes, to determine if they might co-localize in a multimolecular complex, sometimes referred to as a “heparanosome.” It is an attractive hypothesis that, because heparan sulfate synthesis is controlled and mostly sequential, all the enzymes may be present in a supramolecular complex where a core protein is processed in a continuous fashion. Certainly, prior biochemical analysis of microsomal preparations suggests that sulfation events in glycosaminoglycan synthesis are a fast process, complete in a few minutes (Höök et al. 1975). To accomplish these localization experiments, either antibodies against the enzymes need to be prepared or characterized, or cDNAs encoding full-length enzymes as fluorescent fusion proteins can be transfected into cells. Rather few commercial antibodies are available to the many synthetic enzymes that contribute to heparan sulfate assembly. Moreover, some have to be treated with caution. The risk is not only that the antibody, particularly when it is polyclonal, may have unwanted additional activity against cellular components. An additional possibility is that an antibody may cross-react with more than one enzyme, particularly when it may share structural properties that reflect its function. The only completely satisfactory control comprises staining of cells for a specific enzyme in control and knockout cells, usually derived from mice. This should yield no staining and no products in Western blots. In practice, many of the enzymes that contribute to heparan sulfate synthesis have been knocked out in mice. Some, however, such as EXT1, EXT2, NDST1, and HS6ST-1, are embryonic lethal (Lin et al. 2000; Ringvall et al. 2000; Stickens et al. 2005; Sugaya et al. 2008), underscoring the necessity for heparan sulfate in embryonic development. In such cases, cDNA expression in fibroblasts or other cells can be a good way forward. The only risk is that overexpression may lead to “overspill” into cellular compartments where the enzyme is not normally present. In practice, however, we have found that each ectopically expressed transferase only localizes to one Golgi compartment. In some cases, it may be possible to combine immunocytochemistry and ectopic protein expression as a further control, because the two assays should yield an identical result. As a note, it should be remembered that most Golgi enzymes, including all those in heparan sulfate biosynthesis, are type II membrane proteins, so tagging the C-terminus is the only option to retain functionality.

## The EXT Enzymes

**Figure 3.**
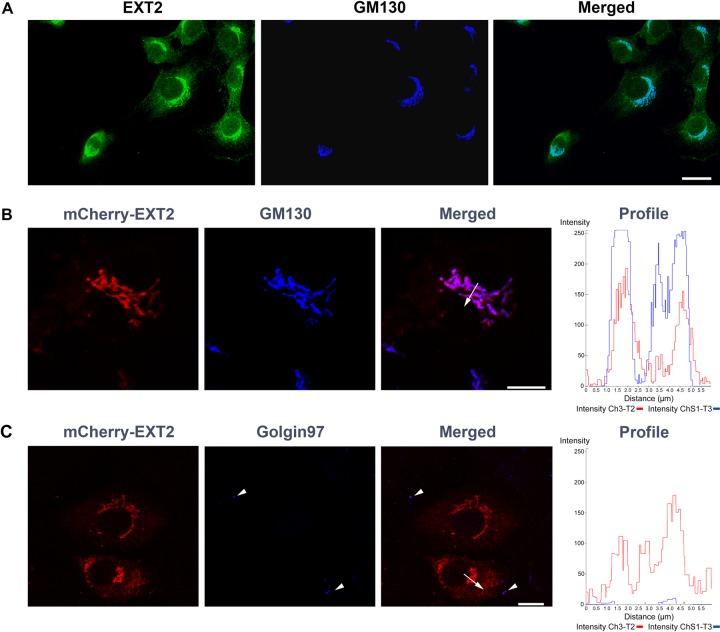
EXT2 protein localized to the cis-Golgi compartment. (A) Endogenous EXT2 protein showed extensive co-localization with the cis-Golgi marker, GM130. Scale bar: 50 µm. (B) The same co-localization was seen for EXT2-mCherry chimeric protein and the cis-Golgi marker. A profile of a confocal microscopy line scan (arrow on the merged image) confirmed the localization of EXT2-mCherry with GM130. (C) Golgin97, a trans-Golgi marker (arrowheads), was not co-distributed with EXT2-mCherry. The confocal microscopy line scan (arrow on the merged image) showed that EXT2-mCherry was located in a different compartment than Golgin97. Scale bars: B, C = 10 µm.

A starting point on the way to mapping heparan sulfate synthetic enzyme mammalian cells has been EXT1 and EXT2. These combine to form the major polymerase and therefore are responsible for the biosynthetic step that immediately follows assembly of the linker tetrasaccharide. These two proteins form a complex (Kobayashi et al. 2000; McCormick et al. 2000), and it transpires that only EXT1 has significant transferase activity, with EXT2 appearing to be a form of chaperone while being homologous in sequence to EXT1 (McCormick et al. 2000; Busse and Kusche-Gullberg 2003). There is evidence that the two EXT proteins cannot localize to the Golgi apparatus independent of each other but become resident in the endoplasmic reticulum (McCormick et al. 2000; Busse et al. 2007). This would be consistent with evidence that mutations in EXT1 or EXT2 genes can lead to hereditary multiple exostoses and that deletion of either gene is lethal in the mouse (Lin et al. 2000; Zak et al. 2002; Stickens et al. 2005). Antibodies against EXT2 strongly suggest a cis-Golgi localization because there is almost perfect colocalization with the GM130 marker by confocal microscopy (Fig. 3A, B). Moreover, precisely the same distribution is seen when a cDNA for EXT2 is expressed in NRK cells, in this case fused to mCherry. This is consistent with a prior observation (McCormick et al. 2000); however, we have also compared staining for other Golgi compartments with that of GM130. In these cases, EXT2 does not co-localize with, for example, α-mannosidase II of the medial compartment or Golgin97 of the trans-Golgi compartment (Fig. 3C).

**Figure 4.**
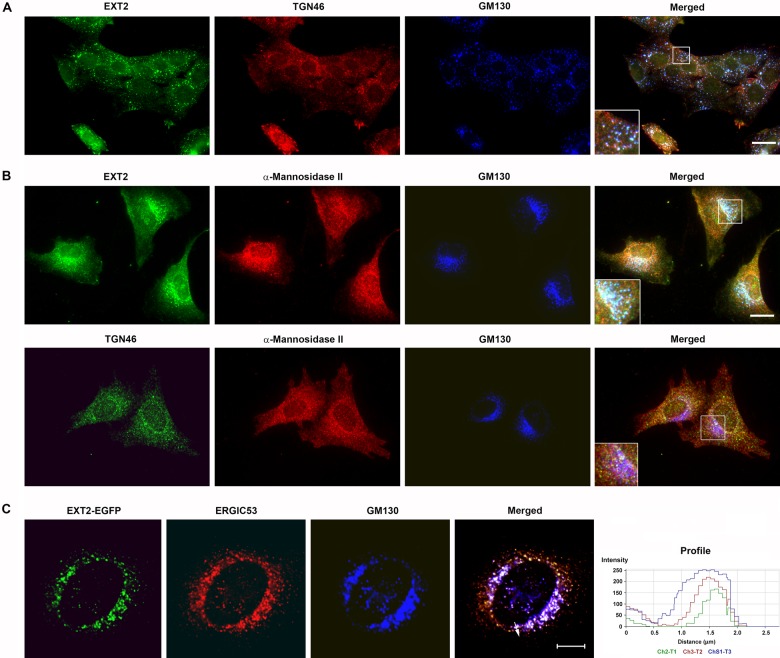
cis-Golgi localization of EXT2 protein was retained in Golgi structures disrupted by drug treatments. Normal rat kidney (NRK) cells were treated with nocodazole (A) or brefeldin A (B, C). (A) Endogenous EXT2 localized with GM130 in ministacks, distinct from TGN46. (B, C) In brefeldin A-treated cells, endogenous (B) and EGFP-chimeric EXT2 (C) underwent retrograde movement with α-mannosidase II and GM130 to the endoplasmic reticulum. TGN46 remained in the trans-Golgi network compartment (B). The retrograde movement of EXT2-EGFP was further supported by confocal line scanning (C). Scale bar: A = 50 µm; B = 25 µm; C = 10 µm.

Further control experiments can be performed to support data from immunocytochemistry and the expression of enzymes ectopically. It is known that when cells are treated with nocodazole, the Golgi apparatus breaks up into “ministacks” (Cole et al. 1996). This underscores that the Golgi is itself a microtubule-dependent organelle for its integrity. However, the interesting feature of this treatment is that the ministacks retain their organization, so that cis, medial, and trans compartments are still discernable (Fig. 4). Moreover, in some cases, the clarity of the compartments under conditions of nocodazole treatment can be very favorable. In Fig. 4A, it is shown that EXT2 is clearly detectable as a cis-compartment component once again, with its distribution coincident with GM130 but distinct from medial- and trans-Golgi markers.

A further well-known effect on the Golgi apparatus is seen when cells are treated with brefeldin A. This compound, a fungal macrocyclic lactone, causes profound disturbance of the Golgi, with cis and medial membranes undergoing retrograde movement back to the endoplasmic reticulum (Klausner et al. 1992). However, trans-Golgi membranes remain distinct, and so brefeldin A treatment can be used to distinctly ascertain whether a component has trans-Golgi localization. In the case of EXT2, its distribution does not follow that of TGN38 or TGN46 markers of the trans-Golgi but rather is disrupted (Fig. 4B, C). This provides some further evidence that EXT enzymes are not associated with the trans-Golgi but with an “earlier” compartment.

## Conclusions and Perspectives

Given the complexity of the fine structure of heparan sulfate yet the conservation of its overall domain organization, there must be some cellular regulation of its synthesis. Many enzymes combine to assemble this polysaccharide on suitable core proteins. Each cell type tends to have a characteristic type of heparan sulfate in terms of domain structure and extent of modification by sulfation. Heparin synthesis by mast cells on the serglycin core protein (Kolset et al. 2012; Rönnberg et al. 2012) is one example of this regulation, in this particular case yielding a highly sulfate product, including precise organization of 3-*O*-sulfation (Casu et al. 1981; Björk and Lindahl 1982). How this is regulated at the molecular level is unknown but clearly more complex than simply regulating the protein expression of component enzymes. In cells that are polarized, with distinct apical and basolateral compartments, it remains to be determined whether each has distinct proteoglycan content. It is also not clear whether the fine structure of heparan sulfate differs on HSPGs from the different compartments. The complexity of Golgi organization in polarized cells is reviewed in this volume (Dick et al. 2012).

In the near future, we shall determine the localization of many of the biosynthetic enzymes, and it will be interesting to see whether all are present together in the same Golgi compartment or spread through various compartments. If they are spread across the Golgi, this will pose further questions regarding how a nascent proteoglycan is appropriately “chaperoned” through the various compartments. However, given the importance of heparan sulfate for multicellular animal life, potential roles in human disease, and the need for better sources of heparin for clinical use, perhaps by in vitro biosynthesis, the organization of the heparan sulfate synthetic machinery is of considerable interest.
